# Remineralization and inactivation of carious lesions treated with silver fluoride in Brazilian children with special healthcare needs

**DOI:** 10.3389/froh.2024.1345156

**Published:** 2024-03-27

**Authors:** Nicoline Potgieter, Viviane Pereira, Roberto Elias, Senda Charone, Sonia Groisman

**Affiliations:** ^1^ Faculty of Dentistry, University of the Western Cape, Bellville, South Africa; ^2^ Mestre em Periodontia-SL, Doutoranda em Odontopediatria, São Leopoldo Mandic, Sao Leopoldo, Brazil; ^3^ Academia Interamericana de Pacientes Especiais, Clinic Department of Brazilian Association of Dentistry from Duue de Cazias, Rio de Janeiro, Brazil; ^4^ Community Dentistry, Federal University of Rio De Janeiro, Rio de Jeaneiro, Brazil

**Keywords:** silver diamine fluoride (SDF), silver fluoride, caries remineralization, caries inactivation, children with special care needs

## Abstract

**Introduction:**

Providing conventional, restorative dental care to children with special healthcare needs (CSHCN) often requires sedation using general anesthesia. Saliva consistency, diet, and oral hygiene practice are different for CSHCN, and limited evidence is available on the efficacy of silver fluoride (SF) for the management of carious lesions for this vulnerable population.

**Methods:**

Parents of CSHCN were educated about silver fluoride as a treatment option for caries. In total, 550 carious lesions from 100 participants were identified and scored according to the Nyvad Caries criteria. A total of 100 lesions with Nyvad scores 1, 2, and 3 were treated with a single application of silver fluoride and observed postoperatively at 1, 3, and 6 weeks.

**Result:**

The results indicate statistically significant (*p* < 0.05) differences in lesion remineralization over the 6-week follow-up period. At the 6-week follow-up, more than 85% of all lesions were remineralized across all children, regardless of condition or original Nyvad score of 1, 2, or 3.

**Conclusion:**

A single application of silver fluoride has demonstrated effectiveness in remineralization and inactivation of carious lesions over 6 weeks among Brazilian CSHCN. Silver fluoride should be considered an option for the management of carious lesions among CSHCN. Further studies are recommended, including larger sample sizes, longer follow-up times, a second application of SF, and different special needs conditions.

## 1 Introduction

Children with special healthcare needs (CSHCN) often experience functional impairment, impacting their oral health, as well as facial growth and development, making conventional dental care more challenging. CSHCN often also have impaired intellectual development and communication skills, and therefore do not have the ability to interpret and express dental pain. It has been reported that CSHCN require more complex restorative treatments as the affected children do not report pain as easily (1). Several investigations have highlighted the difficulties of performing conventional dental care on children with cognitive impairment (2–4).

Providing dental care to CSHCN often requires hospitalization to receive dental treatment under general anesthesia. Minimally invasive dentistry has shifted the focus to remineralization or inactivating caries as an alternative to conventional restorations. The ideal caries management technique for CSHCN should be a simple, non-invasive dental procedure that lasts and that is able to remineralize the tooth surfaces below and around restorations. The atraumatic restorative technique (ART) has proven to be a viable option for some patients; however, the ART procedure still requires moderate cooperation and is considered a temporary solution. As described by Rosenblatt et al. (5), products containing silver fluoride (SF) have become a non-invasive alternative to dental restorations. In vitro studies suggested that silver fluoride regimens also inhibit the growth of *Streptococcus mutans* (4). SF is a new generation of the well-known silver diamine fluoride (SDF) with improved properties. SF is ammonia-free and therefore has less risk for tissue burn and irritation, improved smell, has a more physiological pH, and has more stability, therefore not requiring refrigeration (6). If disease progression can be halted through remineralization/inactivation of carious lesions, it might assist in the overall management and oral health of CSHCN without the need for repeat sedation or general anesthesia appointments.

Therefore, the aim of this study was to evaluate the clinical remineralization and inactivation of active carious lesions among Brazilian CSHCN after being treated with a single application of silver fluoride.

## 2 Materials and methods

Approval from the Ethical Committee was obtained (CEP, Brazilian Association of Dentistry, Duke de Caxias, Rio de Janeiro, Brazil) before the study. The study was conducted at the Brazilian Association of Dentistry Caxias, State of Rio de Janeiro, Brazil.

In total, 125 CSHCN diagnosed with autism spectrum disorder (ASD), Down syndrome (DS), or cerebral palsy (CP) at the age of 7–12 years and their parents were invited to participate in the study. The children had to have at least one carious lesion indicated for conventional restoration [caries limited to dentin, according to Nyvad et al. (7)] (Table 1).

**Table 1:** Description of Nyvad caries criteria (7).

Score	Diagnostic	Criteria
0	Sound	Normal translucency and enamel texture (possible light pigmentation in a sound fissure).
1	Active caries (intact surface)	Enamel surface opaque, no shiny surface, whitish/yellowish enamel surface, rough on probing, usually covered with bacterial plaque. There is no detectable loss of substance. On a smooth surface, the carious lesion is located close to the gingival margin. In pits and fissures, the morphology is intact and the lesion extends along its walls.
2	Active caries (surface discontinued)	Same criteria for score 1. Superficial defect. Localized (microcavity) only in enamel. Absence of soft enamel.
3	Active caries (cavitation)	Enamel/dentin cavitated easily visualized by eye; surface of cavity softened on gentle probing. It might have or not pulp involvement
4	Inactive caries (intact surface)	White, brownish or dark enamel surface. Enamel can became shiny hard and smooth on gentle probing. There is no clinically detectable loss of substance. On a smooth surface, the carious lesion is typically located to some distance from gingival margin. In pits and fissures, an intact morphology is observed with the lesion extending along the cleft walls.
5	Inactive caries (surface discontinuity)	Same criteria for score 4. Superficial defect localized (microcavitaded) in enamel. Without enamel softened on probing.
6	Inactive caries (cavitation)	Enamel/dentin cavity easily visible clinically—cavity can be shiny and hardened on probing with light pressure. Without pulp involvement.
7	Restoration with sound surface	Normal translucency and enamel texture (possible light pigmentation in a sound fissure).
8	Restoration + active caries	Caries active lesion could be cavitated or not
9	Restoration + inactive caries	Caries inactive lesion could be cavitated or not

Parents were informed about SF by making use of the patient information leaflet adapted from the British Society of Paediatric Dentistry (8). The information included the following: use of silver compounds in medicine and dentistry; composition of SF and its mechanism of action; the indications for SF treatment; the procedure of application; effectiveness and possible concerns; and photographs of lesions treated with SF. Written consent was obtained by parents/legal guardians as well as assent from the participating children aged over 12 years. This is the age for obtaining assent for research purposes in Brazil. The parents who did not authorize their child to be in the study or did not accept the terms of consent, still received dental treatment in the conventional away as offered by the facility. All identified children received additional and/or emergency treatment if needed before commencement of the study.

### 2.1 Clinical procedures and follow-up

Active carious lesion from each participant were identified for the study and scored according to the Nyvad system to obtain baseline data (Table 1) (7). All clinical procedures were performed by qualified, experienced single operators and all Nyvad scoring was carried out by an additional single operator. An experienced expert in the Nyvad scoring system, who was calibrated by Bent Nyvad in 2012, trained the principal investigator who conducted all examinations and Nyvad scoring. Interrater reliability between the expert and principal investigator was Kappa = 0.81 and intracalibration of the principal investigator was Kappa was 0.89 (Cohen's Kappa coefficient).

**Figure 1:**
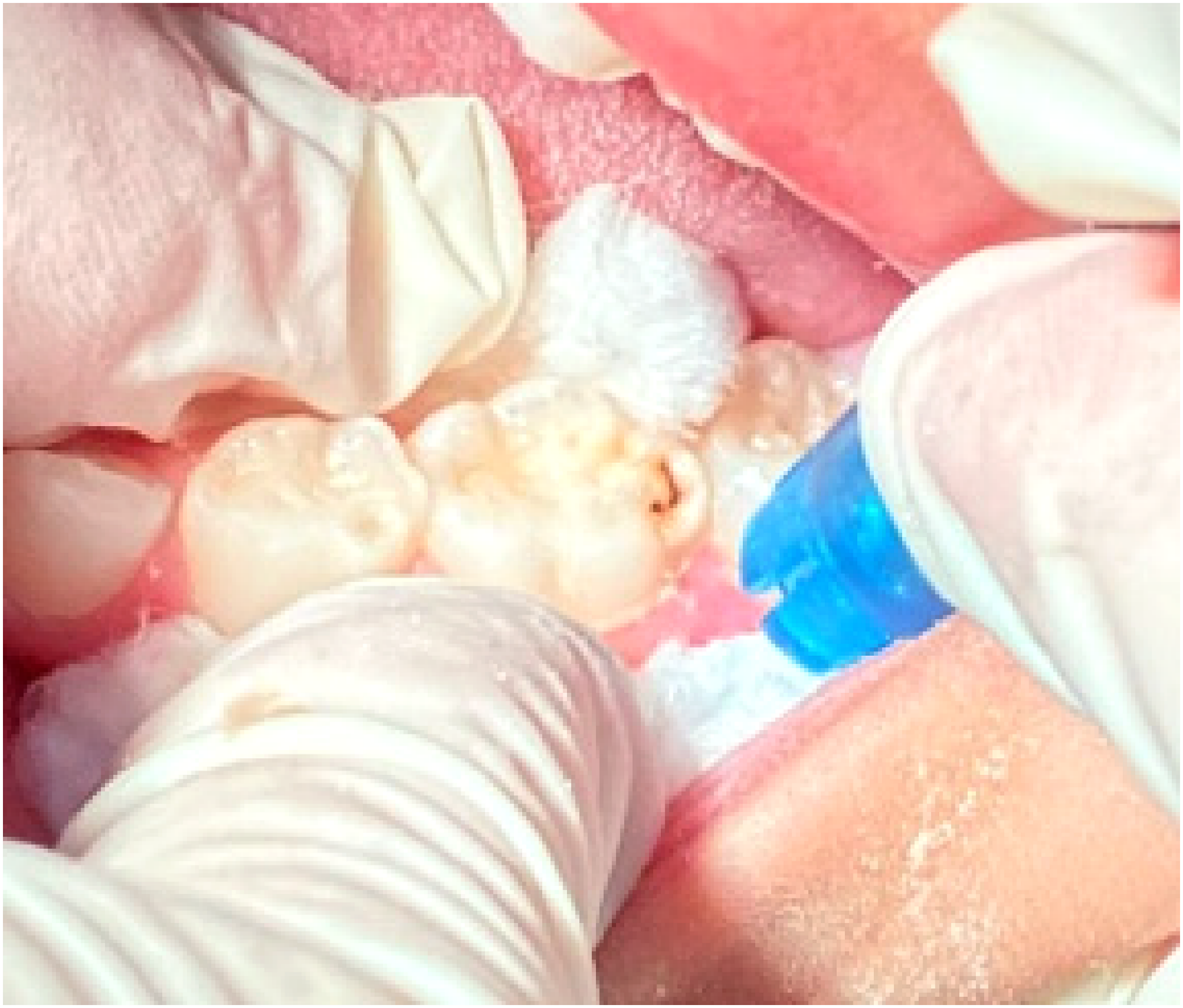
SF being applied in a lesion, with cotton roll isolation.

**Figure 2:**
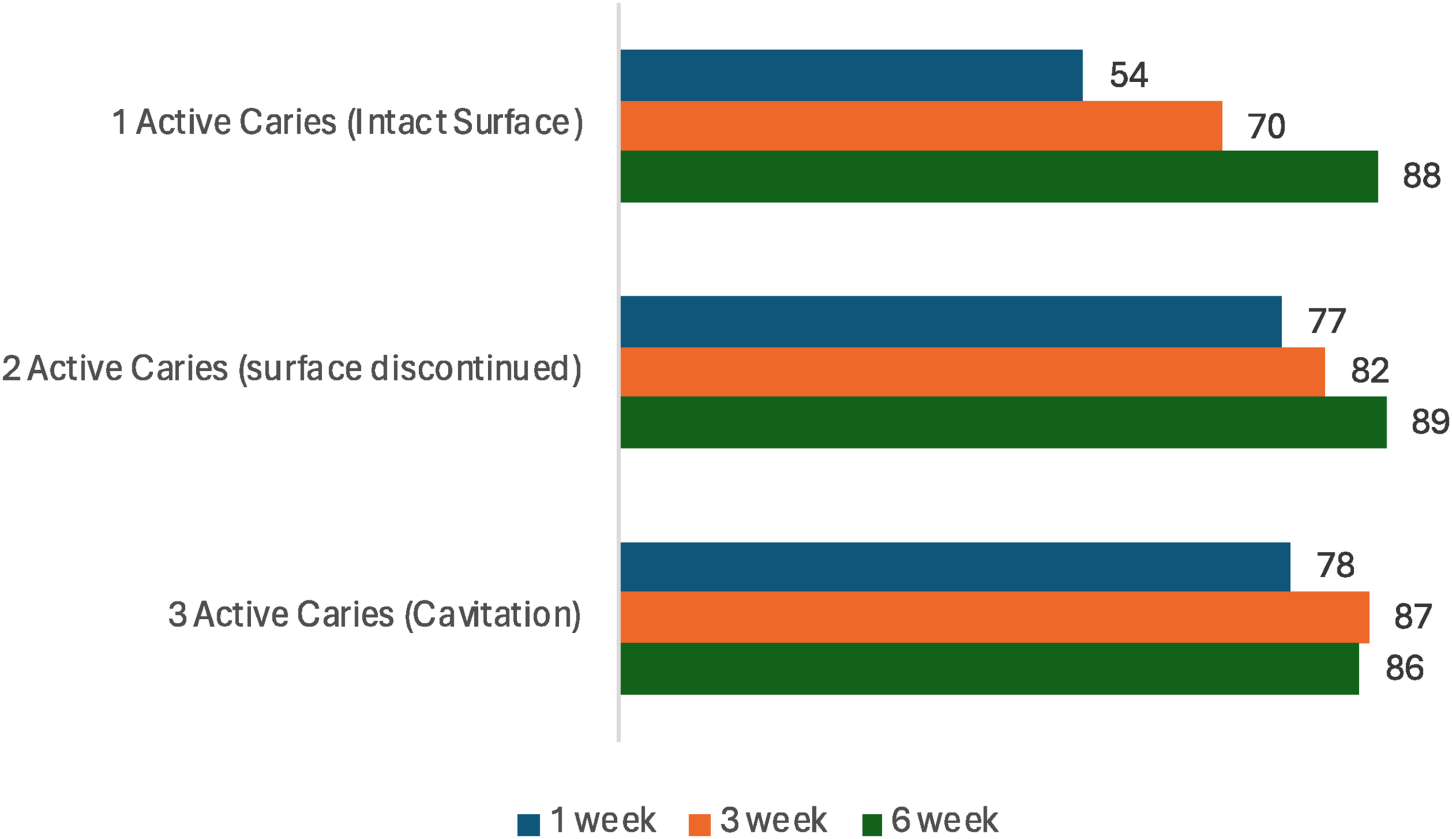
Percentage of lesions that remineralized/inactivated (Nyvad criteria 4–6), after 1, 3 and 6 weeks (*N* = 300 lesions, 100 per score).

After baseline scoring, the SF was applied to the active carious lesions (Nyvad scores 1, 2, or 3) as per the manufacturer's instructions (9):

(1) Isolation: cocoa butter was applied to the lips and surrounding gingival tissues, and care was taken not to inadvertently coat the surfaces of the caries lesions. Isolation of the tooth was achieved with cotton rolls and the lesion was lightly dried with compressed air (Figure 1);
(2) SF application: one drop of SF (Riva Star Aqua, SDI, VIC, Australia, product registration code: D349082 / K172047/ GMDN code 45232 Class IIa) was dispensed into a glass Dappen dish and the microbrush was dipped into the solution. The SF was directly applied to the carious lesion. The lesion was then dabbed with a clean cotton pellet to remove any excess SF, and a gentle flow of compressed air was applied until the medicament was dry. The second step, the application of potassium iodide, was not done in this study, as the aim of this study was remineralization and not limiting discoloration.

No local anesthesia, sedation, or general anesthesia was used during the visit.

Patients were recalled at 1, 3, and 6 weeks after the single application of SF and the lesions were evaluated again according to the Nyvad scoring system at each visit to record remineralization within and around the treated lesions.

### 2.2 Statistical analysis

The results are reported with a descriptive analysis in the form of percentages. Differences in remineralization levels were evaluated with the Friedman test between weeks 1, 3, and 6. Pairwise comparisons were made with the paired Wilcoxon signed-rank test. *p*-Values were adjusted using the Bonferroni multiple testing correction method, and *p* = 0.05 was considered to be statistically significant. The statistical analysis was conducted using R Project 4.3.2 software (R Foundation for Statistical Computing, Vienna, Austria).

## 3 Results

In total, 125 children aged 7–12 years and their parents were invited to participate in the clinical study; however, only 100 children took part in this study; 25 were excluded because they did not obtain consent from their parents/guardians to participate. Of the 100 participating children, 28 had DS, 27 had CP, and 45 had ASD. In total, 550 carious lesions were identified and scored according to the Nyvad criteria, as reported in Table 2.

**Table 2:** Baseline Nyvad scores or lesions observed in 100 children.

Score	Nyvad caries criteria	*N*	Percentage
1	Active caries (intact surface)	171	31.1
2	Active caries (surface discontinued)	111	20.2
3	Active caries (cavitation)	100	18.1
4	Inactive caries (intact surface)	25	4.5
5	Inactive caries (surface discontinuity)	84	15.3
6	Inactive caries (cavitation)	3	0.6
7	Restoration with sound surface	0	0
8	Restoration + Active caries	12	2.2
9	Restoration + Inactive caries	44	8
Total		550	100

Among the lesions, 300 active carious lesions had Nyvad scores of 1 (*n* = 100), 2 (*n* = 100), and 3 (*n* = 100), and were selected and treated with a single application of SF and analyzed for 6 weeks. Teeth with a baseline Nyvad score of 8 would also have been eligible for treatment but had a low prevalence (*n* = 12); therefore, they were excluded from the analysis in this study.

Follow-up evaluations, after a single application of SF at 1, 3, and 6 weeks, are reported in Table 3. The results indicate that the majority of the carious lesions remineralized or inactivated (Nyvad criteria: 4–6 grouped together) throughout the observation period. After 6 weeks, of those carious lesions with a Nyvad score of 1, only 11% did not remineralize; of those lesions with a Nyvad score of 2, only 10% did not remineralize; and of those lesions with a Nyvad score of 3, only 14% did not remineralize. The same pattern was found for all conditions, indicating that one single application of SF was able to promote a quick remineralization/inactivation of the active carious lesions, therefore avoiding progression of the disease. Figure 2 shows the increase of remineralization from week 1 to week 3 and from week 3 to week 6, with the exception of lesions with a Nyvad score of 3 that had a small, non-significant decrease from week 3 to week 6 (from 87% to 86%, i.e., −1 percentage point) but kept a steady increase from week 1 (78% remineralized/inactivated). Statistical tests (Friedman test and paired Wilcoxon signed-rank test) reported statistically significant differences (*p* < 0.05) between the weeks for the total sample and for most of the Nyvad scores in each condition. The most prominent differences have been between week 1 and week 6, also indicating the continuous effect of a single application of SF.

**Table 3:** Baseline and follow-up Nyvad scores of all lesions.

Group	Nyvad caries criteria		Base line	Remineralized Nyvad criteria: 4, 5 or 6			Variation w3-w1	Variation w6-w3	Frieadman *p*-value
	Score	Diagnostic		1 week	3 weeks	6 weeks			
Total (*N* = 100)	1	Active caries (intact surface)	100	54 (54%)^a^	70 (70%)^b^	88 (88%)^c^	16 (16 pp)	18 (18 pp)	<0.001*
	2	Active caries (surface discontinued)	100	77 (77%)^a^	82 (82%)^a^	89 (89%)^b^	5 (5 pp)	7 (7 pp)	<0.001*
	3	Active caries (cavitation)	100	78 (78%)^a^	87 (87%)^b^	86 (86%)^b^	9 (9 pp)	−1 (−1 pp)	<0.001*
	1, 2, 3	Total active carious lesions	300	211 (70%)^a^	244 (81%)^b^	265 (88%)^c^	33 (11 pp)	21 (7 pp)	<0.001*
Autism (*N* = 45)	1	Active caries (intact surface)	45	25 (56%)^a^	32 (71%)^b^	40 (89%)^c^	7 (16 pp)	8 (18 pp)	<0.001*
	2	Active caries (surface discontinued)	45	35 (78%)^a^	37 (82%)^a,b^	40 (89%)^b^	2 (4 pp)	3 (7 pp)	0.022*
	3	Active caries (cavitation)	45	35 (78%)^a^	39 (87%)^a^	39 (87%)^a^	4 (9 pp)	0 (0 pp)	0.072
	1, 2, 3	Total active carious lesions	135	95 (70%)^a^	108 (80%)^b^	119 (88%)^c^	13 (10 pp)	11 (8 pp)	<0.001*
Down Syndrome (*N* = 28)	1	Active caries (intact surface)	28	15 (54%)^a^	19 (68%)^a,b^	24 (86%)^b^	4 (14 pp)	5 (18 pp)	0.001*
	2	Active caries (surface discontinued)	28	21 (75%)^a^	23 (82%)^a^	25 (89%)^a^	2 (7 pp)	2 (7 pp)	0.052
	3	Active caries (cavitation)	28	22 (79%)^a^	24 (86%)^a,b^	24 (86%)^b^	2 (7 pp)	0 (0 pp)	0.001*
	1, 2, 3	Total active carious lesions	84	58 (69%)^a^	66 (79%)^b^	73 (87%)^c^	8 (10 pp)	7 (8 pp)	0.001*
Cerebral Palsy (*N* = 27)	1	Active caries (intact surface)	27	14 (52%)^a^	19 (70%)^a,b^	24 (89%)^c^	5 (19 pp)	5 (19 pp)	<0.001*
	2	Active caries (surface discontinued)	27	21 (78%)^a^	22 (81%)^a^	24 (89%)^a^	1 (4 pp)	2 (7 pp)	0.097
	3	Active caries (cavitation)	27	21 (78%)^a^	24 (89%)^a^	23 (85%)^a^	3 (11 pp)	−1 (−4 pp)	0.097
	1, 2, 3	Total active carious lesions	81	56 (69%)^a^	65 (80%)^b^	71 (88%)^b^	9 (11 pp)	6 (7 pp)	<0.001*

pp, percentage points.

^a,b,c^ Different letters indicate significative differences between observations with the paired Wilcoxon signed-rank test (*p* < 0.05).

* *p* < 0.05.

## 4 Discussion

It has been proven *in vitro* that silver and fluoride ions can remineralize demineralized enamel and dentin (10, 11). Clinical studies have suggested SDF's efficacy in preventing caries in both the primary and permanent dentition and is being widely implemented worldwide for the treatment (arresting) of dental caries (12, 13). SDF has been proven to be safe, effective, patient-centered, timely, efficient, and equitable (14). However, limited scientific evidence is available on the efficacy of SF. Although SDF has an ammonia base and SF is ammonia-free, they contain the same active ingredients—38% silver fluoride and 5% fluoride (SDI, Riva Aqua Product information leaflet)—and the results of this study can therefore be compared with previous studies on SDF. The reason for using SF in this particular study was its improved properties. It was of the authors' opinion that the improved properties would make the treatment more tolerable for children who are sensitive to oral stimulus, taste, or smell. Comparing the patients' acceptance and experience between SDF and SF is an area for future research. Rosenblatt et al. (5) highlighted the need to increase access to care, improve oral health, and ultimately reduce the need for emergency care for CSHCN. However, there is limited evidence on the efficacy of SDF/SF among CSHCN. The authors wanted to explore if the efficacy of SF will differ among CSHCN as they often have different diets, saliva consistency/quantity, and cooperation regarding home oral hygiene practice.

The baseline Nyvad scores of this study (Table 2) report the high number of untreated active carious lesions (Nyvad scores of 1, 2, and 3: *n* = 382) among this group of CSHCN. This prevalence of untreated carious lesions is similar to the results among schoolchildren, as reported by Machiulskiene et al. (15). The possible reasons for untreated carious lesions can include challenges associated with transport, access to care, communication from the child, as well as some practitioners not being comfortable managing CSHCN. Regarding caries treated with restorations, it is notable that no restorations with sound surfaces were reported (Nyvad score 7), 12 restorations had active caries (Nyvad score 8), and 44 restorations presented with inactive caries (Nyvad score 9). These findings suggest that restorations are not stopping disease progression within this population group.

This study did not only focus on the management of cavitated lesions (Nyvad score 3) but also included initial, demineralized lesions (Nyvad score 1, 2) to evaluate the remineralization of these lesions in this vulnerable group of children. Focusing on early intervention and prevention shifts the focus from treating cavities (e.g., fillings) to rather managing the disease and its progression.

Without proper and timely intervention, dental caries and other oral diseases can lead to severe systemic infections, may negatively affect oral health-related quality of life (oral Health Profile-OHRQoL), and are associated with decreased academic performance and school attendance of a child (16, 17). To address the high rate of untreated caries in high-risk populations, the Centers for Disease Control and Prevention recommends school-based sealant programs, which have demonstrated clinical effectiveness and cost effectiveness (18). Furthermore, a review on the effect of SDF in preventing caries in primary dentition showed significant reductions in the development of new caries vs. placebo after 24 months and was not more or less effective after 12 months compared with glass ionomer sealants (19).

The results of this study show that SF has a high prevention rate, as 88% of Nyvad score 1 and 89% of Nyvad score 2 lesions remineralized within 6 weeks (Table 2). No significant differences were found between the different special needs groups as all lesions remineralized (>85%) regardless of the specific condition. These results suggest that SF might be the ideal, cost-effective option for not only treating active carious lesions but also preventing caries among CSHCN. SF requires fewer steps and is less techniques-sensitive than application of fissure sealants, which makes it even more ideal with children with neurological and associated behavioral challenges. It is the authors' opinion that existing sealant programs may benefit from SF as an alternative to fissure sealants or to be used in a combination of placing sealants and treating active carious lesions with SF.

Overall, this study showed inactivation of cavitated lesions at 1 (78%), 3 (87%), and 6 (86%) weeks postoperatively (Table 2). These findings are comparable to those from other controlled clinical trials with longer follow-up times, which indicated no differences in the 6- and 12-month caries inactivation rates comparing SDF vs. atraumatic restorative treatment (19). With limited cooperation associated with CSHCN, even the atraumatic restorative technique can be a challenge. It is important to note that the findings reported in this study are after a single application of SF and that the results might improve with a second application as part of the care plan.

The results of this study indicated that SF could become a key element for prevention and comprehensive management programs that meet the World Health Organization (WHO) Millennium Goals (20). SDF has also recently been added to the WHO’s essential medicine list. The limitations of this study include a small sample size and that it was limited to a follow-up period of 6 weeks after a single application. Further research is recommended to support the findings of this study and to confirm the long-term benefits to this population group.

## 5 Conclusion

A single application of silver fluoride has demonstrated effectiveness in the remineralization and inactivation of carious lesions over 6 weeks among Brazilian CSHCN. Silver fluoride should be considered an option for the management of carious lesions among CSHCN. Further studies are recommended, including larger sample sizes, longer follow-up times, a second application of SF, and different special needs conditions.

## Data Availability

The raw data supporting the conclusions of this article will be made available by the authors, without undue reservation.
